# Inducing and disrupting flow during music performance

**DOI:** 10.3389/fpsyg.2023.1187153

**Published:** 2023-06-02

**Authors:** Julia Zielke, Manuel Anglada-Tort, Jonathan Berger

**Affiliations:** ^1^Center for Computer Research in Music and Acoustics, Stanford University, Stanford, CA, United States; ^2^Department of Psychology, Stanford University, Stanford, CA, United States; ^3^Faculty of Music, University of Oxford, Oxford, United Kingdom

**Keywords:** flow, music, performance, attention, expectation

## Abstract

Flow is defined as a state of total absorption in an activity, involving focused attention, deep engagement, loss of self-conscious awareness, and self-perceived temporal distortion. Musical flow has been associated with enhanced performance, but the bulk of previous research has investigated flow mechanisms using self-report methodology. Thus, little is known about the precise musical features that may induce or disrupt flow. This work aims to consider the experience of flow from a music performance perspective in order to investigate these features and introduces a method of measuring flow in real time. In Study 1, musicians reviewed a self-selected video of themselves performing, noting first, where in the performance they recalled “losing themselves” in the music, and second, where their focused state was interrupted. Thematic analysis of participant flow experiences suggests temporal, dynamic, pitch and timbral dimensions associated with the induction and disruption of flow. In Study 2, musicians were brought into the lab and recorded while performing a self-selected musical composition. Next, participants were asked to estimate the duration of their performance, and to rewatch their recordings to mark those places in which they recalled “losing themselves in the moment.” We found that the proportion of performance time spent in flow significantly correlated with self-reported flow intensity, providing an intrinsic measure of flow and confirming the validity of our method to capture flow states in music performance. We then analyzed the music scores and participants’ performed melodies. The results showed that stepwise motion, repeated sequence, and a lack of disjunct motion are common to flow state entry points, whereas disjunct motion and syncopation are common to flow state exit points. Overall, such initial findings suggest directions that warrant future study and, altogether, they have implications regarding utilizing flow in music performance contexts.

## 1. Introduction

Flow is a state of total absorption in an activity—in other words, the feeling of losing yourself in the moment (Csikszentmihalyi, 1990). It is a psychological state involving effortless and focused attention, deep engagement, loss of self-conscious awareness, a sense of control over the situation, positive affect, temporal distortion (time seems to pass more quickly), and intrinsic motivation (Nakamura and Csikszentmihalyi, 2014). Flow is experienced in a wide variety of activities, including sports—often referred to as being “in the zone” (e.g., Jackson, 1996; Jackson and Csikszentmihalyi, 1999; for a review see Swann et al., 2012), music listening and performance, writing (e.g., Larson, 1988; Perry, 2009), coloring (e.g., Curry and Kasser, 2005; Forkosh and Drake, 2017), playing video games (e.g., Cowley et al., 2008; Michailidis et al., 2018), and other such activities that involve concentration, immediate feedback, clear goals, and a match of situational challenge to personal skill.

The flow state is directly associated with well-being, particularly through its engagement of positive emotions and intrinsic motivation (Csikszentmihalyi, 1990, 2002; Delle Fave et al., 2011; Tse et al., 2020; Loepthien and Leipold, 2021). Moreover, flow by definition is an experiential form of well-being, which has been shown to positively predict declarative well-being—e.g., increased positive mood, job satisfaction, or satisfaction with life (Ilies et al., 2016; Habe et al., 2021). In addition to improving well-being and positive affect, flow may be relevant to coping with mental health challenges, including larger challenges such as the current COVID-19 pandemic (Habe et al., 2021). For instance, Csikszentmihalyi (1990) wrote that the ability to turn a stressful or hopeless situation into an enjoyable and agentic flow experience can help us cope with major life tragedies by translating a threat into an intrinsically-rewarding, goal-oriented challenge. Since flow has been found to be related to wellbeing, the purpose of the current study was to investigate the conditions that seem to support induction into as well as interruption from the flow state. As music activities cultivate flow more often than other activities (Lowis, 2002) and musical flow is understudied (Chirico et al., 2015), we chose to study the conditions for the activation and disruption of the flow state while individuals perform music.

In general, the flow literature has informed which types of musical activities involve flow; namely, composition, listening, and performance (Chirico et al., 2015). For example, previous research has provided preliminary evidence that the goal-oriented creative compositional process and a lack of disruptive thoughts fosters flow, and that negative thoughts can bring one out of the flow state. Crafting a composition through therapeutic songwriting results in high levels of flow (Mac Donald et al., 2006; Baker and Mac Donald, 2013). In fact, songwriting flow has been shown to improve clinical therapeutic outcomes for substance abuse patients (Silverman et al., 2016). Even outside of the music therapeutic context, composition creativity has been found to be positively associated with flow (e.g., Byrne et al., 2003). Additionally, another form of composition that induces flow is improvisation. A small, but growing body of work exists on flow and jazz improvisation. For example, one study by Forbes (2020) investigated improvisational jazz singers’ experiences of flow in the form of anecdotal interviews, finding that (1) flow occurs when the performance is going in the way the performer desires, and is disrupted by the occurrence of negative or self-critical thoughts and that (2) flow in improvisational music is a deeply meaningful, intrinsically rewarding experience. Another study interviewed 18 jazz musicians and found that jazz flow may be induced by the presence of other group members, allowing one to “become one with the group” (Hytönen-Ng, 2013). Beyond interviews, Biasutti and Frezza (2009) found through self-report questionnaires that improvisational flow is positively correlated with musical practice and with anticipation, suggesting that practice improves the fluency necessary to experience flow. Another body of work in this vein has employed investigated improvisational flow through an interactive music composition machine system, which allows feedback during the creative process, one of the components of flow. Past work has shown that composing through this interactive system is conducive to the flow state for musicians (Nijs et al., 2012a,b).

There has been some preliminary work on flow during music listening. One such study had participants either imagine a situation where they performed a musical piece that was challenging and ambitious for them or a situation where they listened to music, finding that music listening was more highly conducive to flow than music performance (Loepthien and Leipold, 2021). Another listening study by Diaz (2013) found that a mindfulness induction allowed participants to experience higher flow, by allowing them to focus more on listening to music. Additionally, in regards to music listening, Ruth et al. (2017) found that flow is positively associated with liking for listened to radio music. However, apart from the three aforementioned studies, there has generally been very little work on flow in the realm of music listening.

The majority of the literature on flow involves music performance (e.g., Sinnamon et al., 2012), and studies on other musical activities often use performance as a reference point. However, as found by a recent review, these performance studies use varied self-report methodologies that may leave ambiguity in distinguishing the flow mechanisms from their confounds (Tan and Sin, 2021). A major finding from the performance literature is that more impediments to flow emerge in the form of the situation exceeding the performers’ skill and in the form of performance anxiety caused by the fear of social judgment. As such, previous work has found a significant negative association between performance anxiety and flow (LeBlanc et al., 1997; Wrigley and Emmerson, 2013; Cohen and Bodner, 2018), and there is some evidence that interventions such as yoga can decrease performance anxiety to increase flow (Khalsa and Cope, 2006; Butzer et al., 2016). Similarly, studies of music performance students found that, since the majority of participants believed that they did not possess sufficient skill to meet the challenge of the performance, they did not find the performance absorbing or intrinsically enjoyable, two of the major conditions of flow (Fullagar et al., 2013; Wrigley and Emmerson, 2013). Interestingly, in the aforementioned study comparing music listening and performance, the authors note that the social-evaluative nature of performance may have impeded flow (Loepthien and Leipold, 2021). However, it is worth noting that the way the authors tested music performance—by inviting participants to think of performing a piece that is ambitious for them (i.e., exceeds their skill level)—already would not likely facilitate flow, since one of the main conditions of flow is a challenge that meets but does not exceed skill level. Indeed, performance context seems to affect the experience of flow vs. performance anxiety. As a further example, Cohen and Bodner (2019) investigated the contextual variables that affect flow and performance anxiety, finding that percussionist professional musicians experienced higher flow and lower performance anxiety than string player professional musicians, and that age is positively associated with flow. Finally, flow during musical performance has physiological correlates—specifically, flow was associated with decreased heart period, decreased blood pressure, and increased respiratory depth and increased heart rate variability (de Manzano et al., 2010). These results suggest that a demanding task like music performance increases activation of the sympathetic nervous system alongside deep breathing, which could be an “indicator of effortless attention and flow” (de Manzano et al., 2010, p. 306).

A recent review found that studies on flow in musical performance primarily have used self-report methods to investigate the psychological conditions of flow (Tan and Sin, 2021). However, the performance paradigms used to induce flow vary greatly. For example, in Fullagar et al.’s (2013) study, participants were asked about their flow during an examination. This led to the authors finding that flow is linked to performance anxiety. However, flow has also been found to be linked to improved performance and effortlessness (de Manzano et al., 2010). This study instead had musicians bring a piece into a non-evaluative lab context and play it multiple times. The striking difference in methodology in the field has led to mixed conclusions about the psychological nature of flow. Another point of interest about the performance literature is the bias towards dispositional flow, rather than state flow (Chirico et al., 2015). Dispositional flow refers to enduring characteristics that make flow more likely to occur, such as having an autotelic personality (Nakamura and Csikszentmihalyi, 2005). State flow, on the other hand, involves real-time external contexts and characteristics that foster flow (e.g., Bakker, 2005). Dispositional flow measures individual differences, from a psychological perspective, though it is less well equipped than state flow to understand external musical factors which may also be contributing to inducement of the flow state. These non-standardized performance paradigms and partiality to dispositional self-report methodologies not only approach flow from a solely psychological lens, but also leave ambiguity in terms of parsing the mechanisms behind musical performance flow. This in turn motivates the present approach of studying the mechanisms underlying flow from a musical analysis perspective.

While there has been some research on what types of musical activities involve flow, there is very little research on the mechanisms that cause such flow to occur. In other words, there is research on when flow occurs, but other studies have not yet empirically examined *how* it occurs. The active process of music performance presents an illustrative opportunity to investigate the flow state through participant-generated music. This methodology allows for the investigation of flow through a different methodology: musical analysis of real time flow moments. There is some previous work on investigating the mechanisms behind flow induction, particularly through the emotional role of music. For example, Marin and Bhattacharya (2013) asked pianists about the valence and arousal of emotions experienced during the flow state, finding that induced high-arousal positively- and negatively-valenced emotions are associated with the experience of flow. However, while this study does take an important look at explaining the *how* behind flow induction, it still approaches the question of flow from a solely psychological perspective, emotions. No study to our knowledge has yet investigated the intramusical aspects that induce musical emotions and thus may induce flow, which is a part of the gap that this study fills. This new disciplinary perspective provides a novel viewpoint to disambiguate mechanisms behind the state of flow.

The aim of this study was to investigate the features that induce and disrupt the flow state. To this end, we conducted two experimental studies to identify the internal and external factors affecting the activation and maintenance of flow through music performance. In Study 1, participants were qualitatively interviewed about a past flow performance experience on the factors that affect when they are in flow. In Study 2, we built upon this paradigm by recording participants’ in-person performances, capturing when they went in and out of flow in real time. Study 2 compliments Study 1’s qualitative approach by providing quantitative support to the conclusions found in Study 1. Overall, these studies identify musical features that are conducive and disruptive to the flow state.

## 2. Study 1

### 2.1. Materials and methods

#### 2.1.1. Participants

Participants were recruited by sending emails to musician listservs at a large West Coast private university inviting them to participate in the study in return for a $20 Amazon gift card. To participate, all participants were asked to provide a pre-recorded performance video where they recalled being in the flow state. Submitted performance videos were recorded on average 3.5 years (SD = 1.7) prior to the study. Retrospective performances were used since Study 1 was launched during the COVID-19 pandemic, decreasing the possibility of more recent performances. Participants were eight college musicians with on average 13.3 years (SD = 5.9 years) of musical experience with different instrumental specialties, including voice, violin, viola, cello, electronics, and electric and acoustic guitar. Three of the participants were male and five were female. Performances include three solo, and five group events. Additionally, six of the performances were in front of a live audience, while two were private events. The average length of performance was 12.5 min (SD = 12.6).

Consent was obtained from all participants and research was approved by the Institutional Review Board at Stanford University.

#### 2.1.2. Procedure

Participants answered open-ended questions through a Qualtrics survey about their performance and flow. Through the survey, participants submitted and reviewed a self-selected, pre-recorded video of themselves performing. They were asked to note where in the performance they recall “losing themselves” in the music (i.e., when they became so absorbed in the music that they stopped being consciously aware of their performance) as well as noting the timestamps where their focused state was interrupted. Participants were then invited to reflect on their performance and comment on any recollections or thoughts for the specific timestamps they provided. Additional demographic questions regarding participants’ performance background and experience were collected to ensure that the participant was well-versed in the performance of their instrument, which may be a precondition to experiencing flow.

### 2.2. Results

The videos and responses were viewed and qualitatively coded for flow conditions at each timestamp where the performer indicated that they were brought in and out of the flow state. A thematic analytic approach of qualitatively identifying overarching flow condition themes was conducted in order to determine what factors participants identified as influencing and disrupting flow in real time. We adapted the methodology delineated by Vaismoradi et al. (2016) in this analysis. The qualitative analysis was performed by the first author, with support from the third author, both of whom have expert music theoretical and performance expertise. The three main stages of theme development in qualitative content and thematic analysis we performed are as follows: (1) Initialization, involving reading responses and highlighting recurrent meaning units, conceptual and relationship coding and looking for abstractions in participants’ accounts, and writing reflective notes on observed patterns; (2) Construction, involving classifying codes by common meaning, comparing links between code patterns to delineate overarching themes, and labeling categories of code patterns; and (3) Rectification, involving cycles of correction to the themes, and relating themes to established knowledge to contextualize the findings in the broader literature. This qualitative analytic approach allowed for the attainment of rich data that suggest both avenues for future research as well as a starting basis for understanding the mechanisms behind reaching and maintaining the flow state. The conditions found to induce flow and the conditions found to disrupt flow will be reported and connected to the existing literature. Flow inductions and disruptions will be grouped by (1) musical conditions and (2) performance conditions.

#### 2.2.1. Flow inductions

**Figure 1:**
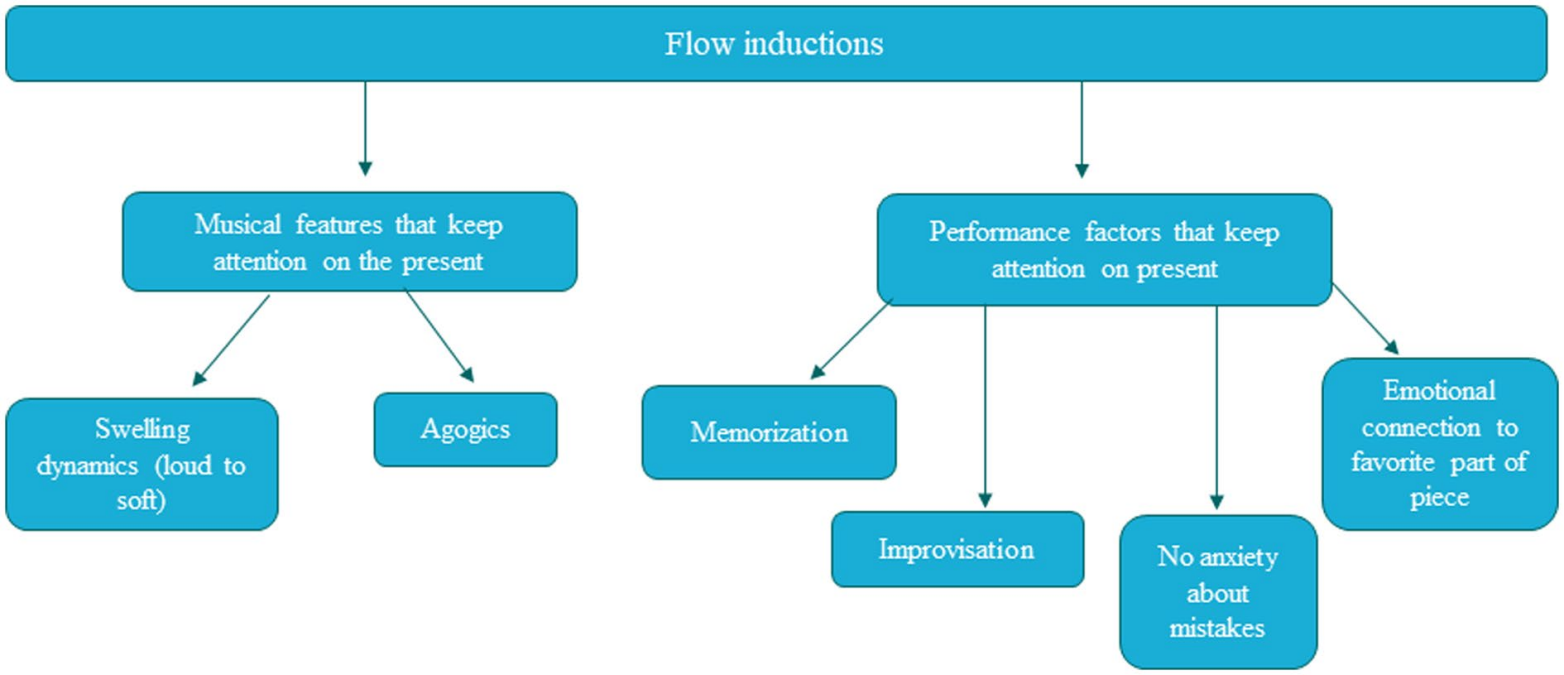
Thematic analysis of factors conducive to flow for participants.

Participant reports indicate that conditions conducive to flow include (1) crescendo and decrescendo dynamics, (2) temporal distortion/agogics, (3) memorization, (4) improvisation, (5) a lack of anxiety and mistakes, and (6) performers’ emotional connection to the section. Figure 1 depicts the different themes identified in the data. The left column shows that these themes fell into two categories: (1) Features of the music that bring attention to the present moment (swelling dynamics, rubato/performance agogics), and (2) Factors that reduce potential for external or performance disruptions (memorization and improvisation, a lack of anxiety or mistakes, emotional connection to the music). Each of these themes will be discussed in turn in relation to the current literature. See Supplementary material for thematic analysis.

##### 2.2.1.1. Musical factors that induce flow

There were two musical factors found to induce flow: Dynamics, and Performance agogics.

Dynamics were a common theme in inducing flow, specifically moments of a musical piece that “swell” (crescendo and then decrescendo, going from forte to piano). For example, one participant noted that their “intensified flow moments … rel [y] on a severe dynamic drop from crescendo to piano.” The existing literature has not examined dynamics specifically in terms of flow, but it has pointed to a relationship between dynamics and emotion, of which is a component of flow. Kamenetsky et al. (1997) found that variations in dynamics resulted in increased perceived emotional expressiveness. Additionally, Gabrielsson and Juslin (1996) found that happy and sad/tender musical expressions involve rapid or slowly increasing volume respectively, as seen in the crescendos (swells) noted by this study’s participants. Such “swelling” dynamics may induce flow, since they can elicit the positive emotion characteristic of flow.

Another identified theme was agogics—places where time and stress are used as emphasis for particular notes. For example, one participant wrote that they “tend to get much more ‘elastic’” with their playing when in flow, referencing their use of agogics. Like dynamics, performance agogics have not been studied in terms of flow. However, Rosenblum (1994) conveys how rubato is associated with emotional expressivity. Therefore, it may be that emotional expressivity through rubato is related to the positive mood characteristic of flow.

##### 2.2.1.2. Performance factors that induce flow

There were four performance factors found to induce flow: Memorization, Improvisation, Having no anxiety about mistakes or technical spots, and one’s Favorite part of piece/emotional connection to section.

Memorization was found to increase the likelihood of experiencing flow. As an example, one participant wrote that they “try to learn [their] music by heart and let it become instinctual,” since doing so helps them with “nerve management” and giving “a moving, heartfelt, and present performance.” Previous studies have found that lower heart rate variability during working memory-and attention-demanding tasks such as performance can indicate lower mental effort, which in turn relates to the effortless and focused attention of flow (Bruya, 2010; de Manzano et al., 2010; Keller et al., 2011) Therefore, reducing the demand on memory in performance may be a mechanism by which memorization increases the likelihood of reaching flow. Additionally, increased practice reduces later performance anxiety (Norton et al., 1978). Thus, the high amount of practice required to memorize a piece may also increase comfort and decrease the likelihood of flow-disrupting mistakes.

Participants also identified improvisation as a factor that induced their flow. In fact, one participant who performed in a group with a dancer said, “I lost myself when my improvised music became somehow one with the dancer.” Some jazz literature discusses improvisatory flow. Mistakes disrupt flow, but improvisation has the special performance capacity of being “able to re-frame mistakes as further fodder for improvisation” (Forbes, 2020, p. 798). In other words, improvisation, as found in the present study, may lessen the impact of mistakes on attention, thus facilitating flow.

Another factor that induced flow was a lack of performance anxiety about mistakes. For instance, one participant wrote, “I did not worry about technique or about producing a mistake … [t] he audience disappeared and it felt as if I were singing through my cello.” Previous literature indicates that flow is more likely to occur when the performance “goes well,” that is, goes in the way the performer desired (Forbes, 2020). Additionally, the same study found that mistakes disrupt attention and flow, and thus the lack of these mistakes in a performance going as desired is conducive to flow. Thus, the theme of a lack of mistakes or anxiety detected in this study fits well with the existing literature.

Finally, emotional expressivity was identified as key to flow. One participant, for example, indicated that their flow “seems to correspond with [their] bias (favorite part of the piece).” Accordingly, previous research has found that performing one’s favorite musical style is more conducive to flow regardless of what that favorite style is (Marin and Bhattacharya, 2013). Thus, the theme that a section being a performer’s favorite makes it more conducive to flow reflects an extension of the previous literature.

#### 2.2.2. Flow disruptions

**Figure 2:**
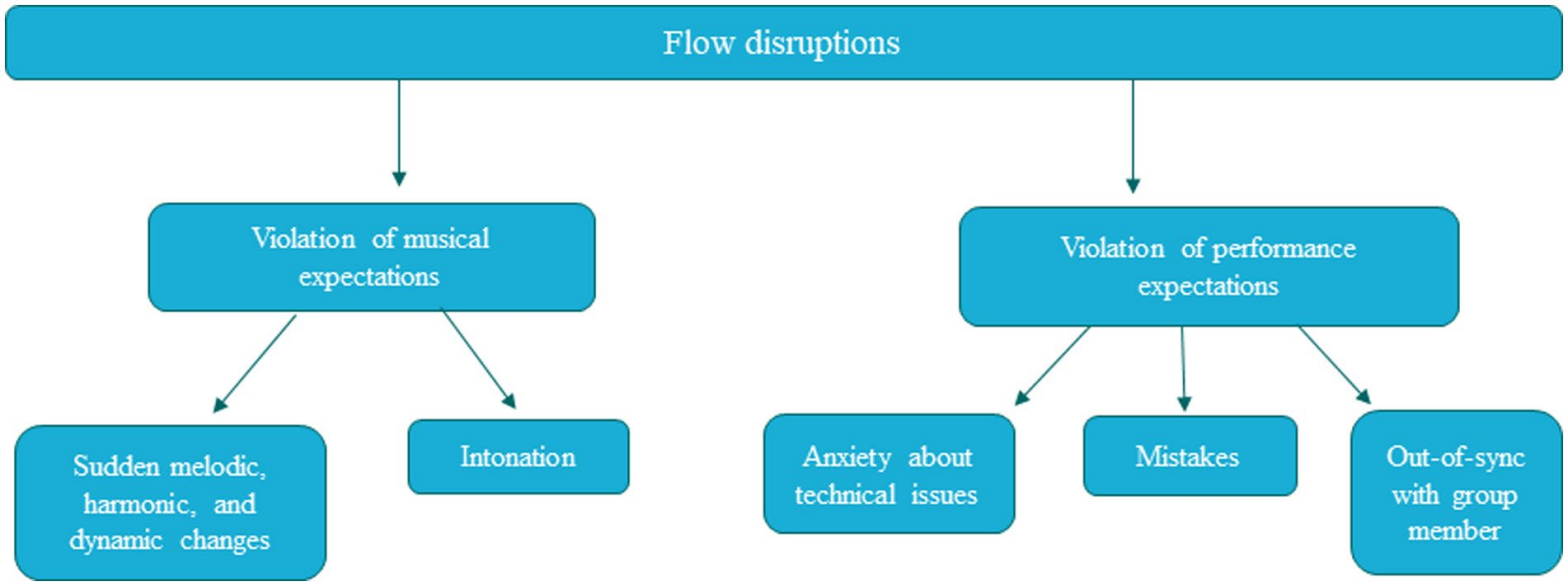
Thematic analysis of factors disruptive to flow for participants.

Participants reported several factors that disrupt flow: (1) Sudden melodic, harmonic, and dynamic changes, (2) intonation/frequency abnormalities, (3) anxiety about physical or technical aspects of performance, (4) mistakes, and (5) feeling out-of-sync with group members. Figure 2 shows the different themes identified in the data. The left column again shows that these themes fell into two categories: (1) Violation of musical expectations (melodic/harmonic/timbre/dynamic changes in the music), and (2) Violation of performance expectations (intonation issues and feeling out-of-sync with group members, mistakes, and anxiety). Each of these themes will be discussed in relation to the current literature. See Supplementary material for thematic analysis.

##### 2.2.2.1. Musical factors that disrupt flow

There were two musical factors found to disrupt flow: Sudden melodic, harmonic, and dynamic changes, and Intonation and being out of tune.

Any surprising melodic, harmonic, and dynamic moments in the music disrupted flow. For example, one participant noted that the moment that took them out of flow was an “abrupt transition from a dimming note to a clash of roaring chords.” While the relationship between flow and melody, harmony, and dynamics has not been directly studied, previous research has indicated a relationship between dynamics, melody, harmony, and attention, of which is a significant factor of the focused flow state. Dynamics are one of the most salient factors that attract attention when listening to music (Macleod et al., 2009), and nonmusicians and musicians are sensitive to dynamics and melody in music (Geringer and Madsen, 1995) as well as harmonic expectations (Loui and Wessel, 2007). Therefore, since dynamics, melody, and harmonic changes seem to attract our attention, unexpected changes in these musical features may draw attention away from the focused state of flow.

Intonation was another factor that was found to disrupt flow. As an example, one person indicated that it was a “slight error in intonation” that brought them “back into awareness.” Previous research has not studied the relationship between intonation and flow specifically, though work on modes of listening has found that intonation is one of the factors that caught listeners’ attention when listening to orchestral performances with focus (Macleod et al., 2009). Thus, it may make sense that mistakes in intonation, as an attention-drawing mechanism, could bring someone out of the flow state.

##### 2.2.2.2. Performance factors that disrupt flow

Having anxiety about upcoming technical or physical aspects disrupted flow. For instance, one participant wrote, “What brought me out of flow was probably my own fear of a difficult part coming up… I came back to reality because I had to convince myself that I could get through it.” Previous studies have found that anxiety over musical accuracy disrupts flow (LeBlanc et al., 1997; Fullagar et al., 2013; Wrigley and Emmerson, 2013). In accordance with this literature, the present study found that anxiety over future technical areas disrupts attention on the present moment and thus disrupts flow.

Making mistakes while performing also was found to draw attention away from flow. One person alluded to this by saying they “flubbed a note” that “forced [them] back into the performance context,” thus disrupting their flow. As aforementioned, mistakes can catch attention and disrupt flow (Forbes, 2020). Therefore, the theme of mistakes disrupting flow aligns with previous work on the dynamics of musical flow.

Disruptions to group dynamics were also found to disrupt the flow state. For example, one participant wrote, “I completely forgot the structure of the piece and was just thrown off when the vocalist entered and just continued soloing instead of moving back into the harmony.” In terms of the literature, group dynamics in flow experiences is a relatively new field of study, though a pilot study by Hart and Di Blasi (2013) found that the combined flow results from getting into the “group groove.” Furthermore, a single mistake by one performer can knock the group out of its “groove,” (Hart and Di Blasi, 2013). Therefore, it may be possible that one performer feels the group groove while others do not. Interestingly, group flow is distinct from individual flow, where group members may or may not be experiencing flow at the same time (Nakamura and Csikszentmihalyi, 2005). Thus, in the case of ensemble performances, disruptions to synchronicity, as found in the present study, may impede flow.

### 2.3. Discussion

Study 1 investigated real-time flow during music performance by having performers retroactively report where in the video of their performance they went in and out of flow. This allowed for the identification of the factors that affect entering and maintaining the flow state. These factors are associated with previous findings in the literature and suggest a number of situations that warrant future study.

It may be that music in line with canonical expectations does not break the performer’s concentration and attention, allowing for the intense focus characteristic of flow. Previous literature has found that music in line with listener temporal expectations enables selective attention (Nobre and van Ede, 2018). In this same vein, our study found that musical features that are in line with expectations are conducive to the flow state, suggesting that the mechanism by which these features modulate flow is through attention. Furthermore, our study found that the activation of the emotional component of flow by musical factors such as dynamics and rubato is conducive to flow. As such, musically-induced emotions during a performance may be conducive to flow experience. This result supports Marin and Bhattacharya’s (2013) finding that self-reported high-arousal emotions are positively associated with the flow state. As such, future research should investigate the affective arousal that the musical features of flow (e.g., dynamics) invoke to understand the affective component of flow from a music feature-analytic perspective. As Marin and Bhattacharya (2013) also found that individual difference variables such as emotional intelligence are associated with flow, it would be interesting for future research to investigate how trait individual differences interact with specific musical features in terms of inducing the affective component of flow. Additionally, future research should investigate if these affective characteristics of music induce flow in additional domains beyond performance, such as music listening. There is no work to our knowledge that identifies dynamics and rubato, both musical features, as aspects conducive to flow. However, previous literature does support that such features modulate attention (Macleod et al., 2009). Thus, the musical features identified in this study may modulate attentional aspects of flow. Lastly, factors that decreased the likelihood of attentional distractions in the form of mistakes (i.e., improvisation and memorization) also made flow more likely to occur. This result supports the literature’s previous findings that flow occurs when a performance “goes well”— in other words, the performance transpires as the performer expects (Forbes, 2020).

In a similar vein, it may be that anything that violates canonical performance or musical expectations (e.g., intonation errors, melodic, harmonic, and dynamic surprises) draws the performer’s attention away from the moment and results in them leaving the flow state. Previous work on violation of expectations indicates that violations of expectations create emotions in music, which may in turn disrupt the emotional component of flow. For instance, Meyer (1956) proposed that musical emotions are formed on the basis of fulfilled or suspended musical expectations, in other words, that the confirmation and violation of musical expectations produces emotions in the listener. Along with the aforementioned findings that the violation of expectations has been shown to attract attention (Janata, 1995; Loui and Wessel, 2007), new or unexpected harmonies lead to specific psychophysiological reactions such as shivers (Sloboda, 1991). Thus, it may be that the violation of expectations can disrupt flow.

Limitations include that this study involved only eight college musician participants, and thus may not be generalizable to other larger contexts, such as non-college musicians or nonmusicians. Further research beyond the anecdotal evidence provided by these eight participants will thus be necessary for generalizability. However, this pilot study was a necessary step to identify flow factors before the paradigm could be applied more generally to a larger population. Additionally, since six of the performances were in front of live audiences, the pressure of performance anxiety may be a confounding factor to our results, since performance anxiety may decrease with higher familiarity and rehearsal with the piece (Sinico et al., 2012) and with higher flow (Marin and Bhattacharya, 2013). However, performance anxiety may still be present regardless of performance expertise as measured in hours of practice (Brugués, 2011), and thus could affect all participants. We note that in this study, the same themes occurred across participants, regardless of musical expertise and performing in front of an audience. One additional limitation is that there was large variance between the length of the submitted recordings (SD = 12.6 min). While we acknowledge that the variation in performance length is a potential confound of this study, we note that our goal was to elicit flow instances in a naturalistic ecologically valid way, which explains why we see this variability. Future work will investigate temporal dynamics as an influence on flow. A further limitation is that the recordings were created on average 3.5 years (SD = 1.7) prior to the study—years had elapsed between the performance and the study, introducing the possibility of participant error in recalling their thoughts and feelings during the performance retrospectively. However, we note that participants reported that their memories of their flow experiences were strong, and that they remembered accurately when they were in flow. For instance, one participant wrote, “In these moments I only thought about expressing to my fullest.” Furthermore, as flow is associated with strong positive emotions (e.g., Croom, 2014; Alexander et al., 2021), and strong emotions are associated with better memory and recall (e.g., Goldstein, 1992; Cahill et al., 1995; Tyng et al., 2017), it may not be surprising that musicians can recall in great detail a past flow experience years later. However, while retrospective recall is a limitation of Study 1, Study 2 addresses this possibility by asking participants to recall when they were in flow directly after performing. Another limitation is that this first study only employed qualitative self-reported interviews, rather than a quantitative data-driven approach. Study 2 will expand on Study 1’s paradigm by investigating flow through an in-person real-time quantitative investigation.

## 3. Study 2

### 3.1. Materials and methods

#### 3.1.1. Participants

Participants were a sample of 25 undergraduate classical musicians who completed the study in return for £30 in cash. Five participants were excluded from the analysis due to either not experiencing flow (1 participant), or failing to provide complete data (4 participants), leaving a final total of 20 participants. Participant mean age was 20.05 years (SD = 1.23 years). In the total sample, 70% were female musicians, and 30% were male. The total instrumental distribution was 40% woodwinds, 25% brass, 15% voice, 10% strings, and 10% keyboard, including flute, oboe, clarinet, alto saxophone, French horn, flugelhorn, trombone, cello, violin, and piano. Participants had been playing their instrument for an average of 11.25 years (SD = 2.31).

Consent was obtained from all participants and research was approved by the Institutional Review Board at Stanford University.

#### 3.1.2. Procedure

Participants were asked to bring a musical composition that they felt comfortable playing to an in-person session. They first read an unrelated article as a distractor task, and then they performed the piece they brought. The distractor task was included in order to reduce subjects’ conscious consideration about whether or not they were in flow while performing. As flow involves focusing attention on the task at hand, bringing attention instead to meta-awareness of whether or not one is in flow could interrupt the flow state. Participants’ performances were recorded on video. After performing, participants filled out a Qualtrics survey including a distractor task (reading comprehension questions), the Flow State Scale Short Form (FSS), and an item asking participants to estimate the duration of their performance. Participants then re-watched their performance video and reported all of the places (timestamps and measure numbers) where they recall “losing themselves in the moment” during their performance. Participants then completed demographic questions and submitted a PDF or picture of the score that they played from.

#### 3.1.3. Measures

##### 3.1.3.1. Flow

We used the Flow State Scale Short Form (FSS, Jackson and Marsh, 1996) to operationalize self-reported flow. Participants were asked to fill out the FSS in relation to how they felt while performing. The FSS is a 10-item scale composed of two subscales—one that measures Absorption (4 items; e.g. “I do not notice time passing”), and one that measures performance Fluency (6 items; e.g. “My thoughts/activities run fluidly and smoothly”). Responses to the items are given on a discrete 7-point Likert scale, ranging from 1 = “Not at all” to 7 = “Very much.” The Cronbach’s alpha for the FSS was α = 0.87, which indicates acceptable reliability (Taber, 2017). The scale is scored by taking the mean of all 10 items to get the total state flow, leading to a continuous score. The mean FSS score across all participants is 4.74 (SD = 0.99). Absorption and Fluency subscores were calculated by taking the mean of the subscale items. The Absorption subscale score had a mean of 4.3 (SD = 0.99) across all participants and an alpha of α = 0.56, indicating low reliability. The Fluency subscale score had a mean of 5.03 (SD = 1.20) across all participants and an alpha of α = 0.91, indicating high reliability.

##### 3.1.3.2. Time distortion

Time distortion is one aspect of flow (Nakamura and Csikszentmihalyi, 2014). In this study, we measured time distortion by asking the participant to estimate how long they spent performing, and we calculated the difference between participants’ time estimate and the length of the recording (how long they actually spent performing).

##### 3.1.3.3. Flow duration

We operationalized flow duration as the proportion of time spent in flow, calculated by dividing the total time spent performing by the time spent in flow (the sum of all of the durations of the participant reported timestamps). A proportion of the total performance time was used instead of a flat value since the reported flow time durations varied greatly (range: 5 to 175 s, mean: 46.9 s).

##### 3.1.3.4. Distribution of pitch intervals

**Figure 3:**
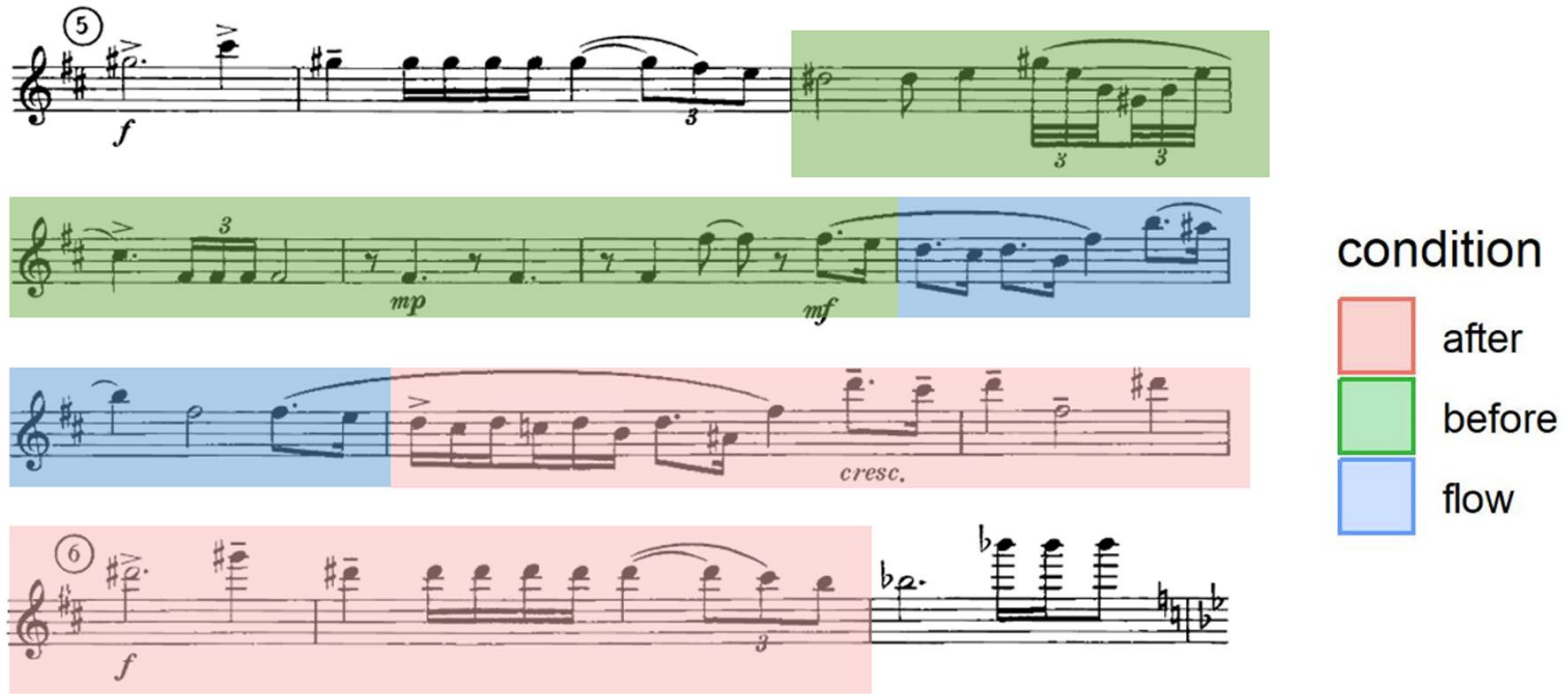
Condition annotated excerpt, from Prokofiev Flute Sonata, mvt 1.

One of the most straightforward ways to examine melodies is by looking at the distribution of pitch intervals (the sequence of two adjacent notes in a melody), which summarizes the frequencies of interval categories in a given set of melodies (Vos and Troost, 1989; Thompson, 2013). Thus, we examined the distribution of pitch intervals in three conditions: the measures that participants indicated being lost in the moment (flow condition), the 4 measures preceding the flow condition (before condition), and the 4 measures following the flow condition (after condition) formed each of 3 conditions for within-excerpt comparison (see Figure 3 for an example). These conditions were each transcribed as score files from the participant-submitted scores through MuseScore 3.6.2, after which they were exported into midi files. The midi files were converted into.csv files using the Python library py_midicsv (Wedde, 2023).

Melodies were represented using MIDI notation, which maps each frequency to a positive integer number (e.g., a middle C4 in a piano keyboard is mapped to the MIDI note number 60). Using MIDI, we can represent melodies using absolute pitch representation (the sequence of MIDI note values that define a melody, e.g., [64, 66, 67, 63, 64]). However, most people represent melodies using relative pitch representation instead (Dowling, 1978), where pitches are expressed relative to each other rather than in absolute terms. Thus, we represented using the sequence of pitch intervals (e.g., [2, 1, −4, 1]). Pitch intervals in each condition were calculated by subtracting each MIDI note value from the preceding one in the sequence of notes making up each melody.

For pianists, only the right hand was transcribed as a melody. Melody flow excerpts reported by participants were excluded if there were not 4 measures on either side of the excerpt that could serve as controls (i.e., if the flow measures overlapped with the before or after of the subsequent or preceding flow excerpt, or if the flow excerpt occurred at the beginning or end of the piece).

### 3.2. Results and discussion

#### 3.2.1. Flow associations

**Figure 4:**
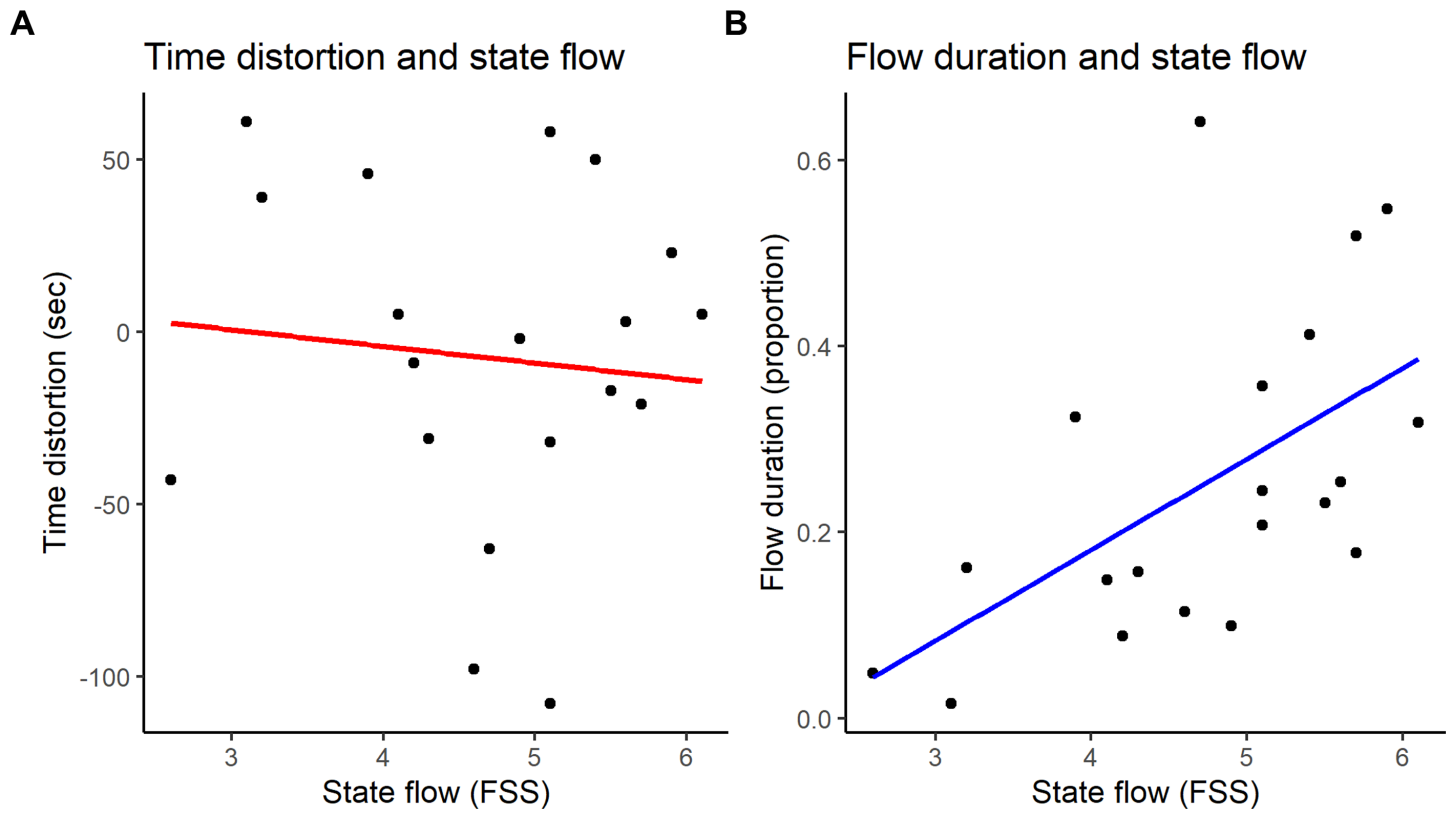
**(A)** State flow is not associated with time distortion (r = −0.100, *p* = 0.67); **(B)** State flow is positively associated with the proportion of time spent in flow (r = 0.562, *p* < 0.01).

To study the relationship between self-reported flow intensity and the temporal dimension of flow, we performed correlation analyses using the stats package in R (R Core Team, 2013). The results of flow associations (duration and time distortion) are shown in Figure 4. We found that self-reported flow state (FSS) and time distortion were not significantly associated [r (18) = −0.100, *p* = 0.67]. However, we found that self-reported flow state (FSS) was significantly associated with the proportion of time spent in flow [r (18) = 0.562, *p* < 0.01]. We then explored further correlations with the FSS subscales (e.g., Engeser and Rheinberg, 2008; Laakasuo et al., 2022) and found that this effect was primarily driven by fluency [r (18) = 0.569, *p* = 0.009]; the subscale absorption was non-significant [r (18) = 0.368, *p* = 0.110]. This finding suggests that the degree, or intensity, of flow in the moment, particularly performance fluency, is associated with the duration of the flow experience. This is consistent with Study 1, which shows that technically challenging parts of the performance disrupt the focus of the flow state, while no anxiety about making mistakes (i.e., performance fluency) facilitates flow. Additionally, the literature points to proficiency in performance facilitating the flow state more so than absorption (e.g., Stupacher, 2019; Spahn et al., 2021); hence, fluency’s prominence in this performance study fits with previous findings as well. This finding additionally confirms that the duration of flow is a valid measure of intrinsic flow experience.

#### 3.2.2. Score analysis

**Figure 5:**
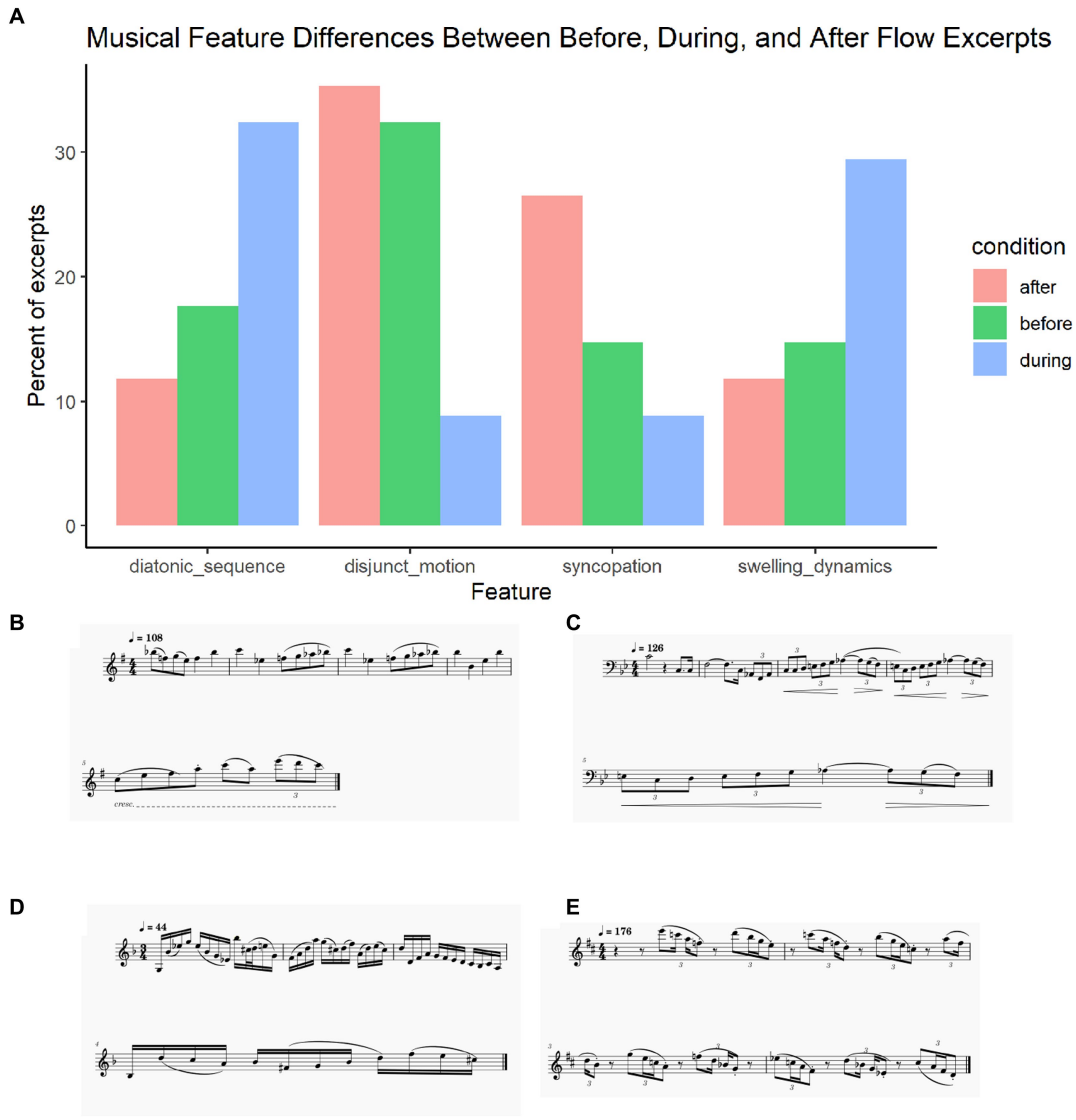
**(A)** Musical feature differences between before, during, and after flow excerpts; **(B)** Repeated sequence during flow, excerpt from Sonatine pour Flûte et Piano by Claude Arrieu; **(C)** Swelling dynamics during flow, excerpt from Concertino for Trombone by Ferdinand David; **(D)** Disjunct motion after flow, excerpt from Concertino for Flugelhorn and Piano, mvt 2 by William Himes; **(E)** Syncopated rhythms after flow, excerpt from Recorda Me by Joe Henderson.

Two music theory experts (the first and third authors) analyzed the scores for the before, during, and after conditions to identify qualitative differences in the music features that are conducive to (during) and disruptive to (after) flow, as compared to a control (before). Results were discussed and confirmed as above (see Figure 5A for a summary of feature prevalence across conditions).

##### 3.2.2.1. Flow inducement

The flow state featured a different pattern of repeated melodic sequence (i.e., the repetition of a passage or motif, often at a higher or lower level of pitch), mainly diatonic sequences, compared to the after condition and before condition. See example in Figure 5B.

Melodic sequence in the literature has long been thought of as something that is in line with musical expectations (e.g., Narmour, 2000). Therefore, it could be that melodic sequence confirms canonical expectations and does not break the performer’s concentration, allowing for the intense focus characteristic of flow, as Study 1 found.

The flow state is also characterized by the presence of dynamic swells with hairpin crescendo decrescendo patterns. This supports the finding from Study 1’s thematic analysis that swelling dynamics conduce flow. See example in Figure 5C.

##### 3.2.2.2. Flow disruption

Additionally, flow was characterized by limited salient disjunct motion (i.e., large interval skips) relative to the before condition and the after condition, which was characterized by more frequent disjunct motion. This makes sense in the context of the finding that large unexpected jumps in pitch catch our attention as measured through event-related brain potentials (ERPs), pulling our attention away from the moment (Stefanics et al., 2009). See example in Figure 5D.

The period after flow also featured more syncopated rhythms than the flow state and the before condition. This finding is contextualized by the literature which has found syncopation to be a way of creating tension (Gomez et al., 2005), which may attract attention away from flow. Supporting this, syncopation, as compared to synchrony, takes more effortful attentional processing, activating additional cortical and subcortical regions of the brain (Mayville et al., 2002). See example in Figure 5E.

#### 3.2.3. Melodic features analysis

Since melodic features were described in the qualitative thematic analysis in Study 1, and melodic distinctions (e.g., lack of disjunct motion) were found to characterize the flow condition relative to after and before flow, we performed melodic features analysis on the excerpts, comparing the flow condition to the before and after conditions as controls.

**Figure 6:**
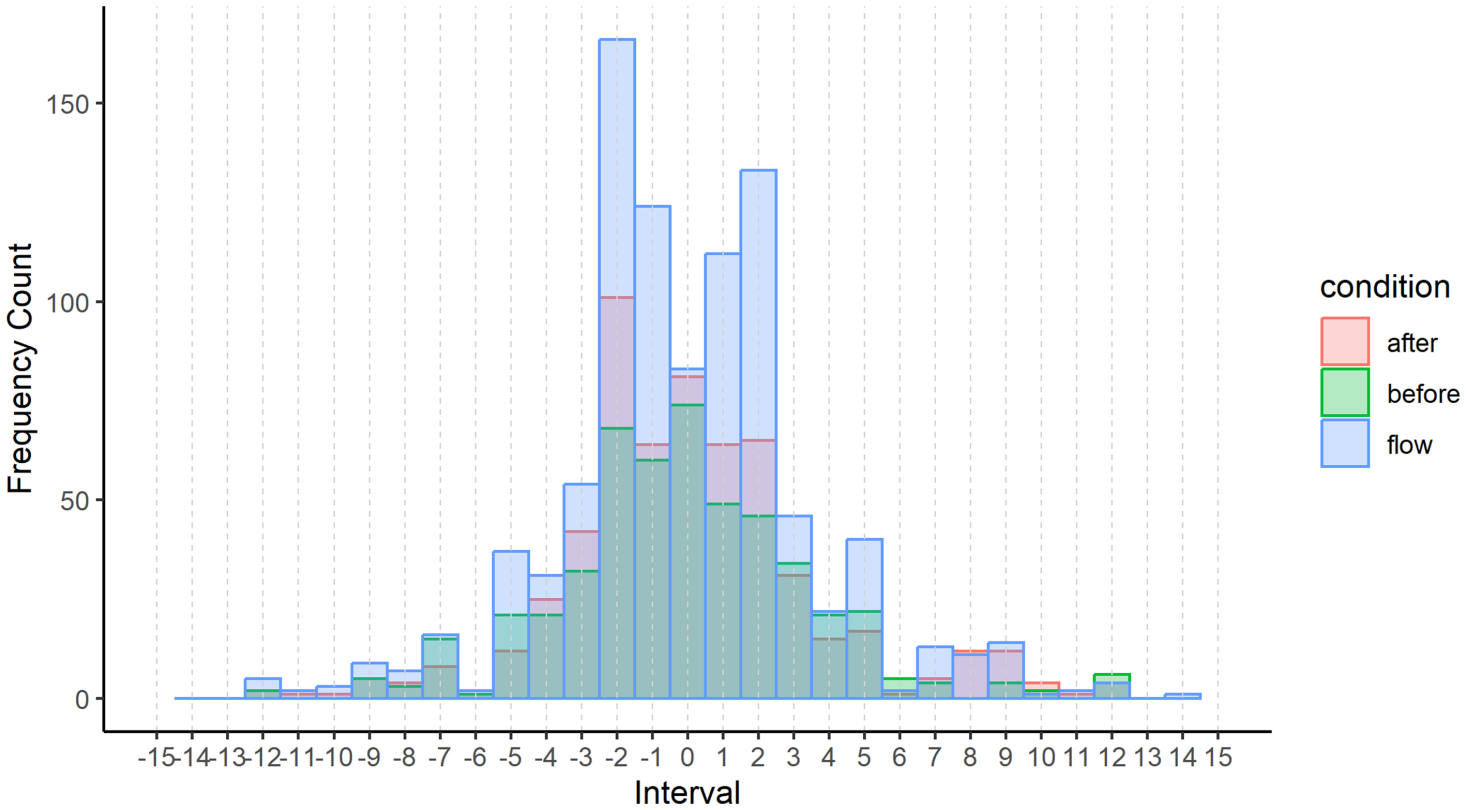
Distribution of pitch intervals across conditions.

As is visible in Figure 6, there is a higher tendency of smaller pitch intervals (< 3 semitones) in the flow condition compared to both before and after conditions. We therefore performed statistical significance tests in R to identify differences in the proportion of small intervals (< 3) across conditions. Since an ANOVA test assumes that the data are normally distributed and assumes homogeneity of variance, and our data were not normally distributed (Shapiro–Wilk test on the ANOVA residuals was significant, W = 0.773, *p* < 0.001) and did not pass homogeneity of variance (Levene test was significant, *F* = 3.23 *p* = 0.04), we used a non-parametric Kruskal-Wallis rank sum test, finding that there was a significant difference between conditions (*p* = 0.035). However, the Kruskal-Wallis test alone does not tell us which groups are different from each other. Therefore, we used a pairwise Wilcox test, finding that flow is significantly different from before flow (*p* = 0.019) and after flow (*p* = 0.040), and before and after flow are not significantly different from each other (*p* = 0.654).

The results revealed that there is a significantly higher proportion of small pitch intervals in the flow condition compared to before and after flow. This indicates a higher degree of stepwise motion in the flow state. This finding provides quantitative support for the finding from the score analysis that flow is less characterized by disjunct motion/interval skips as opposed to the after and before conditions.

In the literature, stepwise motion is widely found to be one of our main melodic expectations when listening to Western music (Cenkerova and Parncutt, 2015). In keeping with traditional musical expectations, computer algorithms that mimic Western composition even use melodic rules such that the generated melody must progress in stepwise motion or, if it jumps, it must continue the stepwise motion from the point where it jumped (e.g., Rader, 1974).

Therefore, stepwise motion confirms our musical expectations, and it follows that breaks in stepwise motion (i.e., disjunct motion) violate our expectations. As such, the finding that the after condition included less stepwise motion indicates that it uses more surprising intervals than the flow condition. Surprising larger intervals and larger melodic jumps have been found to draw attention (Schröger, 1996) and violate expectations (Cuddy and Lunney, 1995). Thus, this melodic analysis result is supported by the literature and additionally provides quantitative support to Study 1’s finding that surprising musical features that violate expectations draw attention and disrupt the flow state.

#### 3.2.4. Discussion

This study is the first to identify musical correlates to the inducement and disruption of the flow state. Study 2 built upon Study 1 by supporting the qualitative themes with musical analysis of the scores and of the melodic features. Specifically, Study 2 found that flow inducement often has a pattern of repeated melodic sequences and swelling dynamics, both of which confirm musical expectations and may draw attention to the emotion of the moment, allowing for the intense focus of the flow state. Flow disruption patterns were found to include syncopated rhythms and disjunct motion, both of which take attention and effortful processing, thus disrupting the focus of the flow state. The melodic features analysis results support the conclusions from Study 1 and the score analysis, finding that the flow state is characterized by stepwise motion, which conforms to musical expectations and thus is conducive to flow. This stepwise motion differs from the disjunct motion that characterizes the measures following flow, and it may be that this forms a surprising change in the melody, akin to the theme expressed by participants in Study 1 that surprising changes in the melody disrupted their flow. All of these results point to a main mechanism of flow: that flow is induced when musical expectations are followed, and disrupted when musical expectations are violated, drawing attention away from being in the moment.

It is interesting to note that the duration of flow is positively associated with flow state. This indicates that the duration of flow has to do with the experienced intensity of state flow. Interestingly, multiple participants in Study 1 indicated a period of more “intense flow” compared to other flow states. For example, one participant wrote that in comparison to “general flow,” “intensified flow moments are especially musically juicy.” Accordingly, this novel flow duration metric may be interesting to consider in further study on flow intensity.

Limitations of this study include that there were a limited number of excerpts and participants, meaning that more research is needed on a larger corpus of excerpts to confirm these musical features’ generalizability beyond this sample. We see potential in future studies to find ways to extend this pattern, for example, by conducting a broadly scalable listening experiment to access a larger sample beyond performing musicians. Additionally, future research should investigate musical features of flow through different avenues, such as music composition.

## 4. General discussion

The present study considered flow during music performance to investigate the musical feature correlates of flow state entry points and exit points. Putting the two studies together, we found that flow inducement involves swelling dynamics, and performance agogics (e.g., rubato) as well as repeated melodic sequences. These features confirm musical expectations and may draw attention to the emotion of the moment, allowing for the intense focus of the flow state. Flow disruption patterns were found to include sudden melodic, harmonic, and dynamic changes, syncopated rhythms, and disjunct motion, which violate expectations and command attention and effortful processing, thus disrupting the focus of the flow state.

Altogether, Study 1 and Study 2 suggest a number of musical-feature situations that warrant future study in the flow literature, as well as propose a mechanism for how musical features affect the state of flow: through musical expectations’ effect on our attention and focus. This flow mechanism could also apply to other flow activities such as sports (e.g., Jackson and Csikszentmihalyi, 1999), or playing video games (e.g., Cowley et al., 2008). Auditory expectations in general have evolutionary roots, allowing organisms to identify and evaluate noisy or ambiguous stimuli for danger (see Pearce and Wiggins, 2012). Given the nature of auditory expectations as an evolutionarily-important skill, this expectation-attention flow mechanism may be particularly interesting to study with respect to activities with a strong auditory component like music or sports. However, the broader framework of expectation-attention may apply to other situations (e.g., writing, coloring) as another component related to the challenge-skill balance of flow. It may be, for instance, that we have expectations of meeting the challenge-skill balance during performance, and when those expectations are violated, we break out of the flow state. Future research can investigate this hypothesis further.

Previous studies on musical flow primarily employ self-report methodology (Moneta, 2012), and, accordingly, there is little research on a music-driven explanation of flow—this study is the first, to our knowledge, that investigates flow from a musical feature-analytic perspective rather than a solely psychological perspective (i.e., using music as a medium to study flow more generally rather than as a source of flow). This study therefore is also the first to look into why music is one of the activities that induces flow most frequently (Lowis, 2002).

Flow has been studied using interviews, questionnaires, and experience-sampling methods, and while the latter allows for the attainment of a large corpus of flow moments, it necessarily interrupts the flow experience, a notorious problem in the flow literature (Nakamura and Csikszentmihalyi, 2009). The present studies address this limitation by introducing a method of measuring real-time flow: recording a performance and viewing it immediately afterwards affords a measure of when flow occurred during the moment without interrupting the flow state as it occurs. This is also an important strength of our study given that much of flow research is performed through self-report questionnaires asking participants to recall a distant time or imagine a hypothetical experience (Moneta, 2012). This study presents a new methodological paradigm for investigating musical flow that complements self-report inventories. Accordingly, another strength of the present studies is that, together, they provide a dual and complementary qualitative (Study 1) and quantitative (Study 2) approach, providing methodologically rich data for the novel study of musical features in flow. Additionally, we present a novel method of measuring the duration of flow: quantifying the proportion of time spent in flow. This is, importantly, a more intrinsic measure, thus less subject to potential self-report biases. This measure could be used in future studies on flow, even beyond music performance. For instance, flow duration could be measured in a flow listening study to identify which songs induce flow for more of their relative runtime.

Given that we measured state flow in the present study, future studies could also include a trait flow measure such as the Flow Proneness Questionnaire (SFPQ; Ullén et al., 2012), in order to investigate how the duration of state flow during a specific music performance associates with the trait-level flow experiences during musical activities (Butkovic et al., 2015). It may additionally prove interesting to explore how state flow maps onto trait flow in future work on musical flow.

One limitation of this study is that we did not operationalize the differences between regular and intense flow, a distinction alluded to by multiple participants in Study 1. This is an open question reflected in the literature as well, reported by qualitative interviews as the difference between shallow and deep flow (Moneta, 2010). Future research can investigate this difference further in how it relates to flow intensity, as measured by a participant’s FSS score. This study found that spending a higher proportion of time in flow is related to higher FSS, or higher reported flow intensity. This novel measure of flow could be used in future research to investigate the qualitative difference between shallow and deep flow more quantitatively.

Another limitation of this study is that it only investigates flow from the performance perspective. As Chirico et al. (2015) note in their review, music performance may be less conducive to flow than other modes of music engagement (i.e., composition and listening), due to its association with performance anxiety (e.g., Fullagar et al., 2013; Cohen and Bodner, 2018). Therefore, one important next step for future research is to investigate the musical features of flow through other forms of music engagement, such as listening. Music listening also allows the study of musical features of flow to expand beyond the study of musicians (as in the present studies) to the general population.

The present study paves the way for future directions in flow research from the music-analytic perspective. For example, future studies can employ a similar method of analyzing further musical features (e.g., frequency and amplitude of recordings themselves) of self-reported flow through music information retrieval, on a larger sample of participants. Future work also should investigate the direct relationship between musical features that induce and disrupt flow and affect—for instance by asking the participant to report both affect and flow while rewatching their performance or listening to a song recording. Additionally, future work should explore other cultural domains and traditions of music beyond the realm of Western classical music. It will be interesting and imperative to investigate if these musical features of flow are culture-specific or universal aspects of human musical experience.

Overall, this work has important implications for music performance research. Through a novel music-analytic perspective of performance flow, we identified musical features that induce the flow state and musical features that disrupt it. This paradigm paves a new pathway for research into the underlying mechanisms of flow, as well as how they interact with other features of performance, such as attention and affect. Additionally, the findings of this study have practical performance implications. For example, flow inducement features could be implemented in practical performance situations by choosing pieces for performance that involve these features or by adding them in as practical interpretations (e.g., adding dynamic and rubato expression), in order to increase the likelihood of attaining the flow state during performance, and decrease the likelihood of disrupting it. A possible further extension of this work is in music composition: Utilizing these features in composition could potentially create more flow-inducing pieces for classical or film music, or even for the pop music industry. Additionally, listening to performances and recordings involving these musical features could potentially help nonmusicians tap into flow. Future research can investigate these potential implications in detail. In essence, maintaining the flow state through paying attention to musical inducement features and minimizing flow disruption features may better allow us to tap into the positive emotions and intrinsic motivation characteristic of flow, which may, in turn, act as an avenue through which music engagement can improve wellbeing.

## Data availability statement

The datasets presented in this study can be found in online repositories. The names of the repository/repositories and accession number(s) can be found below: https://github.com/jzielk/Inducing-and-disrupting-flow.

## Ethics statement

The studies involving human participants were reviewed and approved by the Institutional Review Board, Stanford University. The ethics committee waived the requirement of written informed consent for participation.

## Author contributions

JZ carried out the experiment. JZ carried out the analyses with support from MA-T and JB. JZ wrote the manuscript. MA-T helped supervise the project. JZ and JB conceived the original idea. JB supervised the project. All authors contributed to the article and approved the submitted version.

## Funding

Funding came from the School of Humanities and Sciences Faculty Research Fund, Stanford University. Funding was used to compensate participants.

## Conflict of interest

The authors declare that the research was conducted in the absence of any commercial or financial relationships that could be construed as a potential conflict of interest.

## Publisher’s note

All claims expressed in this article are solely those of the authors and do not necessarily represent those of their affiliated organizations, or those of the publisher, the editors and the reviewers. Any product that may be evaluated in this article, or claim that may be made by its manufacturer, is not guaranteed or endorsed by the publisher.
