# Supplementary material for: Inducing and disrupting flow during music performance
Source: Front Psychol. 2023 Jun 2;14:1187153. doi: 10.3389/fpsyg.2023.1187153 (PMC10272888; doi:10.3389/fpsyg.2023.1187153)
Supplement: Supplementary file 1 [file Data_Sheet_1.docx]

***Supplementary Material***

Inducing and disrupting flow during music performance

**Julia Zielke^*^, Manuel Anglada-Tort, Jonathan Berger**

*** Correspondence:** Corresponding Author: [jzielke@stanford.edu](mailto:jzielke@stanford.edu)

# Supplementary Tables

**Supplementary Table 1.** Thematic Analysis of Flow Inductions

| Overall Concepts | Identified Themes | Examples |
| --- | --- | --- |
| **(1) Features of the music that bring attention to the present moment** | *Dynamics, specifically crescendos followed by decrescendos* | “The intensified flow moments …rel[y] on a severe dynamic drop from crescendo to piano and a special/particular increase in vibrato.”  “it begins louder and drops to very quiet and tender material in closure.”  “My flow …is intensified during the most dramatic/emotional/dynamic/contrasting moments of the piece.” |
|  | *Performance agogics/rubato* | “I tend to get much more “elastic” with my playing when I’m in a flow state than when I’m not.” |
| **(2) Factors that reduce potential for external or performance disruptions and keep attention on the present moment** | *Memorization* | “I try to learn my music by heart and let it become instinctual. This helps with my nerve management, and moreover, to try to give a moving, heartfelt, and present performance.” |
|  | *Improvisation* | “I lost myself when my improvised music became somehow one with the dancer. … The music and the choreography of the piece was based on a structured improvisation. Therefore, we had planned that at certain times what style of music will be accompanying the dancer.”  “Flow feels very different for me when improvising” |
|  | *No anxiety about mistakes or technical spots* | “It felt as if I had championed the technique needed to produce the sound I wanted to make; I didn’t worry about technique or about producing a mistake. The audience disappeared and it felt as if I were singing through my cello.”  “What I recall from entering flow was that I felt I lost control of my fingers. They were just moving and I didn’t really have to think too hard about it – it felt second-nature. The audience and their eyes seemed to disappear.” |
|  | *Favorite part of piece/emotional connection to section* | “My flow seems to correspond with my bias (favorite part of the piece) and is intensified during the most dramatic/emotional/dynamic/contrasting moments of the piece.”  “This is one of my favorite sections of any piece to play, because I love the fluidity of it in my hands.” |

**Supplementary Table 2.** Thematic Analysis of Flow Disruptions

| Overall Concepts | Identified Themes | Examples |
| --- | --- | --- |
| **(1) Violation of musical expectations** | *Sudden melodic, harmonic, and dynamic changes* | “It’s an abrupt transition from a dimming note to a clash of roaring chords. The ending grabs the attention of the player and the audience because of its power and grandiosity.”  “The octave brought me back into conscious awareness (interrupted flow) ... the new musical gesture of the exposed octave.” |
|  | *Intonation / being out of tune* | “beyond the new musical gesture of the exposed octave, the slight error in intonation brought me back into awareness.”  “Something that did affect me at one point was noticing an out of tune string, which made me worried to fix it in time before the end of a tutti phrase.”  “My D string was audibly sharp, which led me to think more about how I would adjust to the string than about the music. I don’t remember exactly where, but you might be able to see me try to mess with the string just between the fingerboard and the pegs during the cadenza. I was feeling irritated here because I couldn’t focus and enjoy what was going on in the moment.” |
| **(2) Violation of performance expectations** | *Anxiety or diverting thoughts about technical or physical issues* | “I tend to get stressed (do I remember what comes next?) or irritated (why is this out of tune?) if something mechanically wrong happens mid-performance, which takes me out of flow.”  “There is one arpeggio that you have to switch your left-hand fingering halfway  through – always knocked me out of the zone. It is also near the end of that arpeggio  passage so I mentally prepare for the change”  “What brought me out of flow was probably my own fear of a difficult part coming up. Even though, I practiced this piece a ton of times, I came back to reality because I had to convince myself that I could get through it.”  “I forgot what I had to play next and was kind of panicking.” |
|  | *Mistakes* | “I also wasn’t able to find the previous sounds that I had used for when she had reached that moment - - it was here that I came back to the performance.”  “I flubbed a note that would normally make me go back and work the section again, so it forced me back into the performance context. Notably, when I came out of that flow state, my performance got noticeably jerkier, with more missed notes.” |
|  | *Out-of-sync with group member* | “I realized my music is not really in conversation with the dance.”  “I completely forgot the structure of the piece and was just thrown off when the vocalist entered and just continued soloing instead of moving back into the harmony.”  “Flow feels … very different when playing in a group than when playing by myself. I find it a lot harder when I’m in a group.” |

# Supplementary Questionnaire

**Supplementary Questionnaire 1.** Study 1 Qualtrics Questions

Description Flow is a sense of "losing yourself" while performing (i.e., when you became so absorbed in the music that you stopped being consciously aware of your performance). Think of a time when you experienced that state while performing.

Instructions Please upload a video of a performance where you experienced flow to YouTube (a pre-existing video is recommended). Make sure that the YouTube video is set to Public or Unlisted (not Private). If you need help changing the video privacy settings, see this [link](https://support.google.com/youtube/answer/157177?hl=en&amp;co=GENIE.Platform%3DDesktop) for a step-by-step guide.

YouTubeLink Then, submit the YouTube link to your performance video here:

________________________________________________________________

Privacy If you submitted an Unlisted YouTube link, do you wish for it to remain private?

- Yes (1)
- No (2)

Comp Name of composition and composer:

________________________________________________________________

________________________________________________________________

________________________________________________________________

________________________________________________________________

________________________________________________________________

End of Block: Block 1

Start of Block: Block 2

Instructions In this section, you will answer questions about the performance video you submitted. Please read the questions first and then rewatch your video in order to answer them.

TimestampsEnter Please list the time(s) when you entered flow, that is, when you lost conscious awareness of what you were playing (in minutes and seconds):

________________________________________________________________

________________________________________________________________

________________________________________________________________

________________________________________________________________

________________________________________________________________

TimestampsDisrupt Please list the time(s) when your flow was disrupted (in minutes and seconds):

________________________________________________________________

________________________________________________________________

________________________________________________________________

________________________________________________________________

________________________________________________________________

Observations Please list any comments or observations you have about those moments:

________________________________________________________________

________________________________________________________________

________________________________________________________________

________________________________________________________________

________________________________________________________________

Comments Do you have any additional comments about your performance?

________________________________________________________________

________________________________________________________________

________________________________________________________________

________________________________________________________________

________________________________________________________________

End of Block: Block 2

Start of Block: Block 3

Demographics This section will ask you demographic questions. Please answer them to the best of your knowledge.

YearsPlayed How long had you been playing this instrument at the time of the recording (in years)?

________________________________________________________________

PerformanceFrequency How regularly did you perform in public at the time of the recording?

________________________________________________________________

________________________________________________________________

________________________________________________________________

________________________________________________________________

________________________________________________________________

ReadMusic Did you read music? If so, did you learn your music by heart?

________________________________________________________________

________________________________________________________________

________________________________________________________________

________________________________________________________________

________________________________________________________________

End of Block: Block 3
